# Unity makes strength: multi-wire technique for bailout sheath upsizing during endovascular revascularisations

**DOI:** 10.1186/s42155-025-00643-x

**Published:** 2026-05-04

**Authors:** Lorenzo Patrone, Gianmarco Falcone, Emiliano Chisci, Azzurra Guidotti, Isabelle Van Herzeele, Stefano Michelagnoli

**Affiliations:** 1 Vascular and Endovascular Surgery Unit, San Giovanni di Dio Hospital, Florence, Italy; 2 Vascular and Interventional Radiology Unit, Department of Radiology, Careggi University Hospital, Florence, Italy; 3 Department of Thoracic and Vascular Surgery, Ghent University Hospital, Ghent, Belgium

**Keywords:** Endovascular intervention, Multi-wire technique, Sheath upsizing, Guidewire preservation, Vascular access, Bailout strategy

## Abstract

The use of a 4-F platform is increasingly used during peripheral arterial interventions, with most of the devices now being compatible with 0.018″ and 0.014″ wires. Unexpected complications/procedural steps during such procedures can require sudden sheath upsize. In this occasion, to maintain the original low-profile guidewire(s), usually positioned across the target lesion(s), can be extremely important. Hereby we describe a technique to safely allow bailout sheath upsizing without losing access to the target lesion(s), which can be applied in a various number of clinical scenarios.

## Introduction

Endovascular recanalizations are becoming more technically demanding due to the increasingly calcific disease pattern and the concomitant evolution of revascularization strategies. Several devices are nowadays compatible with 0.018″ or 0.014″ guidewires and able to pass through a 4-F sheath. This low-profile working access is increasingly being used to diminish access-related complications [1–3], but sometimes an unexpected upgrade of the sheath may be required. This paper describes a novel technique to safely upgrade the introducer sheath size while maintaining the 0.014/0.018’’ guidewire (GW) already in place across the lesion.

## Technique

By keeping the original GW(s) in position, either one additional 0.018″ GW (preferably a high-support stainless-steel–shaft wire, to maximise column strength and pushability during sheath advancement) or two additional 0.014″ GWs (preferably labelled as “high support”) can be introduced in parallel through the same sheath as distally as possible to achieve maximum support. Compared with nitinol-shaft wires, stainless-steel–shaft 0.018″ wires provide higher axial support and stiffness which is desirable for bailout upsizing in calcified or tortuous anatomy; nitinol wires may be used if needed but can offer less support. This can be done directly through the sheath valve or by introducing them over a 4-F catheter previously passed over the original wire. Next, the original sheath is safely removed over these GWs and a new 0.035″ compatible larger sheath is introduced over the same multi-wire platform. Although a 0.035″ guidewire is not used, the 0.014″/0.018″ wires act as a composite stiff rail, improving dilator tracking during insertion of the 0.035″-compatible sheath. Once this is done, the additional GW(s) can be removed. A 4-F sheath can accommodate up to five 0.014″ and two 0.018″ GWs in total [4], which supports the feasibility of adding adjunct wires without losing the original access. In the present bailout upsizing manoeuvre, however, we typically add only one 0.018″ or two 0.014″ high-support wires in addition to the in-situ wire(s), and the adjunct GW(s) are removed once the larger sheath is in place. The choice of the extra-GW(s) to be inserted varies based on the ones already available on the table.

**Fig. 1:**
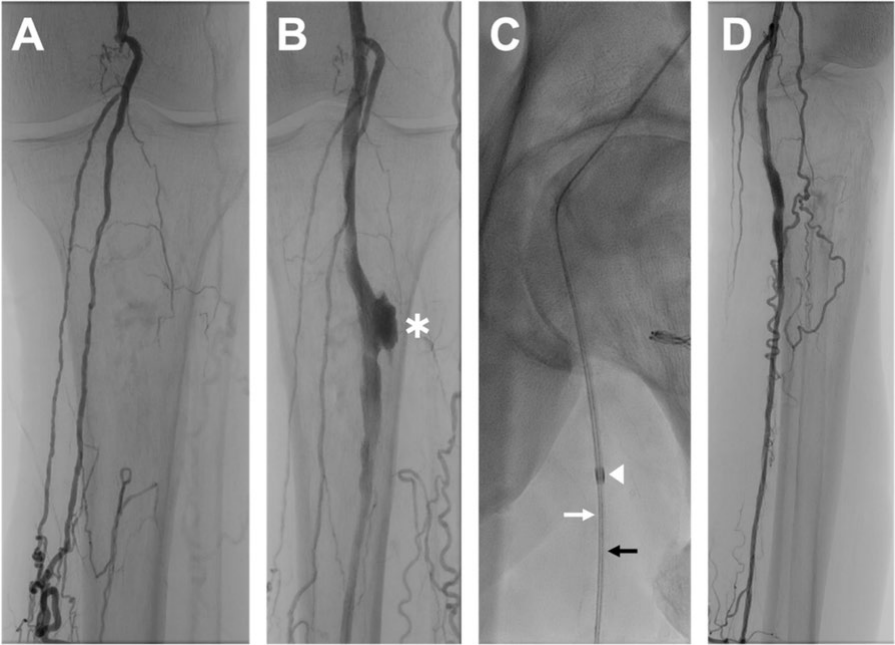
An 83-year-old patient, affected by left lower limb severe rest pain, presented with an atherosclerotic/post actinic chronic occlusion of the popliteal artery extending to the proximal tibial vessels (**A**). This extremely stubborn lesion ruptured during inflation of a 5 mm balloon (Advance 18LP balloon, COOK, Bloomington, IN, USA) with sudden formation of a large pseudoaneurysm (arrow) (**B**). Decision was made to deploy an 0.018″ compatible 6 × 100 cm covered stent across it. A 4F catheter was inserted over the original guidewire (Gladius MG PV ES, Asahi Intecc USA Inc., Irvine, CA, USA) (black arrow) and an additional 0.018″ guidewire (Command ES, Abbott, Chicago, IL, USA) already present on the table (white arrow), was passed through the same catheter down into the proximal posterior tibial artery. Next, the 4 F sheath (Cordis, Hialeah, FL, USA) was removed and a 6-F sheath (Cordis, Hialeah, FL, USA) (white arrowhead) was smoothly inserted over the two 0.018″ guidewires (**C**). The additional 0.018″ guidewire was then removed. A 6 × 100 mm Viabahn stent (Viabahn, W. L. Gore & Associates, Flagstaff, AZ, USA) was deployed with successful exclusion of the large pseudoaneurysm (**D**)

This technique can be extremely useful in different scenarios when to keep the original 0.018″/0.014″ wire(s) in place is key, especially in case of vessel rupture or post angioplasty dissection, where to remove the original wire could lead to loss of lesion access: examples can be bailout covered stenting (Fig. 1), tibial kissing stenting while maintaining the two original 0.014″ GWs in position, sudden use of atherectomy or thrombo-aspiration devices or the additional insertion of a snare to retrieve a balloon/stent fractured over the GW.

## Conclusion

This multi-wire technique for bailout sheath upsizing allows maintenance of the original 0.018″ or 0.014″ GW(s) in their convenient position, increasing intra-procedural safety and potentially reducing total procedural time.

## Data Availability

Not applicable.

## Abbreviation

GW: Guidewire
